# Proof of stability of an RSV Controlled Human Infection Model challenge agent

**DOI:** 10.1186/s12985-024-02386-y

**Published:** 2024-05-15

**Authors:** Sandra Verstraelen, Dirk Roymans, An Jacobs, Karen Hollanders, Sylvie Remy, Dirk Jochmans, Jelle Klein, Tini Grauwet

**Affiliations:** 1 Environmental Intelligence Unit, Flemish Institute for Technological Research (VITO), Industriezone Vlasmeer 7, Mol, 2400 Belgium; 2 DNS Life Sciences Consulting, Brandhoefstraat 63, Turnhout, 2300 Belgium; 3 Rega Institute for Medical Research, Herestraat 49, Leuven, 3000 Belgium; 4 Clinical Pharmacology Unit, SGS, Drie Eikenstraat 655, Edegem, 2650 Belgium

**Keywords:** RSV-NICA, CHIM, TCID_50_, Plaque, Titration, MucilAir™, Infectivity/attack rate

## Abstract

In 2018, SGS Belgium NV developed RSV-NICA (Respiratory Syncytial Virus-Nasobronchial Infective Challenge Agent), an RSV type A challenge agent for use in RSV Controlled Human Infection Model (CHIM) studies.

It is widely recognized that the stability of RSV can be influenced by a variety of environmental parameters, such as temperature and pH. Consequently, our objective was to evaluate the stability of the viral titer of RSV-NICA following five years of controlled storage and to determine the uniformity of the viral titers across different vials of a GMP-qualified batch of RSV-NICA. In addition, we examined the capacity of RSV-NICA to infect human primary airway epithelial cells (MucilAir™), the principal target cells of RSV, and evaluated the influence of single and recurrent freeze–thaw cycles on the infectious viral titer of the challenge agent.

The aliquoted RSV-NICA virus stock was subjected to standard virological and molecular methods to gather data on the titer and consistency of the viral titer contained within 24 representative vials of the stock. Our findings illustrate that over a span of five years of cryo-storage, the infectious viral titer in 75% of the tested vials exhibited a comparable average infectious viral titer (4.75 ± 0.06 vs 4.99 ± 0.11; *p*-value = 0.14). A considerable reduction down to an undetectable level of infectious virus was observed in the remaining vials. RSV-NICA demonstrated its capacity to effectively infect differentiated human airway epithelial cells, with active virus replication detected in these cells through increasing RSV genome copy number over time. Virus tropism for ciliated cells was suggested by the inhibition of cilia beating coupled with an increase in viral RNA titers. No discernable impact on membrane barrier function of the epithelial lung tissues nor cytotoxicity was detected. Pooling of vials with infectious titers > 4.0 log_10_ TCID_50_/ml and freeze-thawing of these combined vials showed no deterioration of the infectious titer. Furthermore, pooling and re-aliquoting of vials spanning the entire range of viral titers (including vials with undetectable infectious virus) along with subjecting the vials to three repeated freeze–thaw cycles did not result in a decrease of the infectious titers in the tested vials.

Taken together, our findings indicate that long-term cryo-storage of vials containing RSV-NICA challenge agent may influence the infectious viral titer of the virus, leading to a decrease in the homogeneity of this titer throughout the challenge stock. However, our study also demonstrates that when heterogeneity of the infectious titer of an RSV stock is observed, rounds of pooling, re-aliquoting and subsequent re-titration serve as an effective method not only to restore the homogeneity of the infectious titer of an RSV-A stock, but also to optimize patient-safety, scientific and operational aspects of viral inoculation of study participants during at least the period of one RSV CHIM trial. RSV-NICA is a stable, suitable CHIM challenge agent that can be utilized in efficacy trials for RSV vaccines and antiviral entities.

**Supplementary Information:**

The online version contains supplementary material available at 10.1186/s12985-024-02386-y.

## Introduction

The COVID-19 pandemic has catalyzed an urgency to refine methods for vaccine and antivirals development. Over 200 vaccines targeting SARS-CoV-2 have been rapidly developed and tested across various stages of (pre-)clinical development within a few short years [9]. The evaluation of these vaccines for safety and efficacy has necessitated significant time and resources, particularly during late-stage clinical development. Consequently, early proof-of-concept trials on newly developed entities have been identified as a priority, not only for SARS-CoV-2, but also for other respiratory viruses of pandemic potential or high medical concern. A prime example is RSV, for which there remains a persistent need for additional vaccines and therapeutic treatments.

While RSV infection typically results in mild upper respiratory tract infections with symptoms akin to the common cold, it is a significant cause of serious respiratory illness, often resulting in bronchiolitis, pneumonia and mortality in patients of various ages [4, 13, 15, 16]. The burden of RSV infection is most pronounced in children under 5 years of age [15]. In this demographic, it was estimated that in 2019, there were 33.0 million RSV-associated acute lower respiratory infection episodes, 3.6 million hospital admissions due to RSV-associated acute lower respiratory infections, 26,300 in-hospital deaths from RSV-associated acute lower respiratory infection, and 101,400 overall deaths attributable to RSV [13]. Other individuals at high risk of developing serious lower respiratory tract infections include those with compromised immunity, underlying lung and heart defects, and the elderly [12, 16]. In adults aged ≥ 50 years from industrialized countries, RSV is estimated to cause 1.5 million episodes of illness, with approximately 14.5% requiring hospitalization and 1.6% resulting in death [17]. Despite the recent approval of the first vaccines -Arexvy and Abrysvo- along with the extended half-life human monoclonal antibody Nirsevimab, there remains a need for additional preventive and therapeutic measures to reduce the disease burden across all high-risk patients.

Early phase pharmacological trials primarily assess the safety and pharmacokinetics of an investigational medicinal product (IMP). However, CHIM trials additionally offer the opportunity to demonstrate the efficacy of IMPs such as vaccines, monoclonal antibodies, or small molecule antivirals, thereby providing early proof-of-concept in clinical development. CHIM studies are designed to simulate the progression of natural infective disease(s), the effects of the disease-causing agent on the human body, and the responses to drugs (*e.g.* antibiotics, vaccines, antivirals) and other interventions. In a CHIM study, a well-characterized strain of pathogen (in this case RSV) is administered at a controlled dose (before or after administration of IMP) and via a specific route to carefully selected, healthy volunteers under highly controlled quarantine conditions within a Human Challenge Unit. This approach affords developers a quicker understanding of the mechanism-of-action and potential efficacy of the IMP at an early stage of development. In addition, this method can serve as a substitute for large-scale phase 3 studies, which may be challenging or impossible under certain circumstances. This is particularly relevant for new 2nd and 3rd generation COVID vaccines and for other infections, such as *Bordetella pertussis*, where there is already a high vaccination coverage in the population.

For RSV, one challenge agent is commercially available and has been used for many years. The Memphis 37 wild type RSV-A strain was isolated from a 4-month-old infant in 2001 [10] and has since been used in numerous CHIM trials, proving its utility in determining the efficacy of new entities. A recent example is the approval of Pfizer´s RSV vaccin Abrysvo for adults over 60 years old, which was tested in a phase 2a CHIM trial [18]. Although this strain is still viable, the symptomology rate is estimated to be approximately 41% based on Pfizer´s CHIM trial to 61.5% based on the most recent CHIM trial with the Ad26.RSV.preF from Janssen Pharmaceuticals. In general, the field would benefit from a broader range of RSV challenge agents to provide the possibility for cross-validation.

The objectives of the experiments reported herein were twofold. First, we aimed to determine the current viral (infectious) titer in representative vials of an RSV-NICA challenge agent and to ascertain the homogeneity of the viral titer present in the different vials of the RSV-NICA batch, thereby assessing the stability of the viral titer of RSV-NICA over a period of five years of cryo-storage. Second, we sought to investigate the potential of RSV-NICA to infect human airway epithelial cells, the primary target cells of the virus, and to assess the capacity of the virus to replicate in these cells. Lastly, we endeavored to examine the impact of a single or repeated freeze–thaw cycle on the infectious viral titer of pooled and resuspended vials of RSV-NICA stock and determine if it was possible to restore and maintain homogeneous infectious titers.

## Materials and methods

### Isolation and preparation of RSV-NICA challenge agent

The RSV type A virus was originally isolated from a nasopharyngeal (NP) swab, collected from an otherwise healthy 15-month female infant in November 2015. The infant was part of a Community Surveillance (non-IMP) trial sponsored by SGS, which had the approval of the Ethics Committee of Ziekenhuis Network Antwerpen (ZNA) and Universitair Ziekenhuis (UZ) Leuven. The purpose of this trial was to collect samples from individuals displaying symptoms indicative of an RSV infection. At the time of enrolment, the infant exhibited typical RSV-like illness symptoms. The presence of RSV was confirmed via a Biofire™ polymerase chain reaction, with no other respiratory pathogens identified at that time. Following a two-month period, all symptoms had been resolved, indicating a full recovery of the subject.

A seed stock was prepared from the isolated virus through culture on a commercial MRC-5 cell line (ATCC CCL-171). This seed stock was subsequently inoculated on MRC-5 cells and stabilized in an 30% sucrose solution to generate the final product under Good Manufacturing Processes (GMP) conditions, in compliance with current US and EU regulations. All batches have been stabilized in this solution and was therefore consistent over the different studies performed. Two batches of RSV-NICA stock were produced, and viral titers from individual vials were determined using TCID_50_ measurement (refer to Table 1).

**Table 1:** TCID_50_ titer determination of randomly selected RSV-NICA vials

Box ID	Sample ID	TCID_50_/ml (log_10_)	Average TCID_50_/ml ± SEM	Box ID	Sample ID	TCID_50_/ml (log_10_)	Average TCID_50_/ml ± SEM
**I**	**I1**	4,80	4.93 ± 0.26	**VII**	**VII1**	2,35	3.22 ± 0.76
	**I2**	5,67			**VII2**	2,52	
	**I3**	4,45			**VII3**	2,52	
	**I4**	4,80			**VII4**	5,49	
**II**	**II1**	4,80	5.23 ± 0.15	**VIII**	**VIII1**	2,35	2.35 ± 0.00
	**II2**	5,32			**VIII2**	2,35	
	**II3**	5,32			**VIII3**	2,35	
	**II4**	5,49			**VIII4**	2,35	
**III**	**III1**	3,57	4.45 ± 0.34	**IX**	**IX1**	2,52	3.05 ± 0.64
	**III2**	4,27			**IX2**	2,35	
	**III3**	5,15			**IX3**	4,97	
	**III4**	4,80			**IX4**	2,35	
**IV**	**IV1**	5,49	5.41 ± 0.21	**X**	**X1**	5,49	5.67 ± 0.07
	**IV2**	4,80			**X2**	5,67	
	**IV3**	5,67			**X3**	5,67	
	**IV4**	5,67			**X4**	5,84	
**V**	**V1**	2,52	2.44 ± 0.05	**XI**	**XI1**	3,40	2.83 ± 0.24
	**V2**	2,35			**XI2**	2,35	
	**V3**	2,52			**XI3**	2,52	
	**V4**	2,35			**XI4**	3,05	
**VI**	**VI1**	2,35	2.35 ± 0.00	**XII**	**XII1**	5,15	4.23 ± 0.64
	**VI2**	2,35			**XII2**	4,80	
	**VI3**	2,35			**XII3**	2,35	
	**VI4**	2,35			**XII4**	4,62	

In 2018, the virus demonstrated appropriate infectivity in vitro and in vivo, exhibiting a profile typical of contemporary wild-type RSV A viruses. This included the absence of overt pathogenesis or excessive virulence in cotton rats infected with RSV-NICA during a GLP-certified animal challenge study (unpublished data).

Freeze-thawing cycles involved snap freezing vials on dry ice before storage in a -80 °C freezer. Thawing of vials was achieved by placing the vials on ice.

### *TCID*_*50*_* determination*

The TCID_50_ assay was performed as outlined in [19]. Briefly, HEp2 cells (ATCC CCL-23) grown in Dulbecco’s Minimum Essential Medium (DMEM; Gibco/Thermo Fisher Scientific, Waltham, USA) supplemented with 10% fetal bovine serum (FBS, Tico Europe, Amstelveen, The Netherlands), 2 mM L-glutamine (Gibco/Thermo Fisher Scientific) and 1% Non-Essential Amino Acids (NEAA; Gibco/Thermo Fisher Scientific) were seeded (5 × 10^3^ cells/well) in HEp2 medium in 96-well plates (Greiner Bio-One, Vilvoorde, Belgium) and grown until reaching 80–90% confluence. In one plate, three samples and one positive control virus of a known titer (RSV long Strain, ATCC VR-26) were grown. In another 96-well plate, 1:10 serial dilutions of different RSV-NICA samples or positive control virus were prepared in triplicates in HEp2 infection medium (DMEM supplemented with 2% FBS, 2 mM L-glutamine and 1% NEAA). HEp2 cells were then infected with 25 μL/well of the virus dilutions by a 2 h (h) incubation at 37 °C and 5% CO_2_. After incubation, 75 μl of HEp2 infection media was added per well and the plates were again incubated for 4–5 days in the incubator. Lastly, the culture medium was discarded and cells were stained with a 1% crystal violet solution for 15–30 min (min). After washing, plates were scored independently by 2 lab technicians. The TCID_50_/ml was calculated using the Spearman and Kärber method [14]. Titration of the infectious virus in 2018 of individual vials of RSV-NICA was determined by TCID_50_ measurement as referred to in de Waal et al. [6] at Viroclinics Biosciences (Schaijk, The Netherlands).

### Plaque titration assay

The plaque titration assay was performed as described in Van den [20]. Briefly, Vero cells grown in Minimum Essential Medium (MEM,Gibco/Thermo Fisher Scientific) supplemented with 10% FBS and 2 mM L-glutamine were seeded (3.4 × 10^5^ cells/well) in 6-well plates and grown until 80–90% confluence. Cells were then infected with 500 μL of the tenfold serially diluted RSV-NICA virus stocks in phosphate buffered saline (PBS; Thermo Fisher Scientific) by a 1 h incubation at room temperature under gentle rocking of the plates. The viral inoculum was aspirated and followed by addition of 2 mL/well of plaque-agarose growth medium (DMEM/1.5% agarose containing 4% sodium bicarbonate 1% penicillin–streptomycin 12 mL 2 mM L-glutamine, 5% of FBS, adjusted to pH 7.4 with 1 M NaOH maintained at 42 °C). Plates were placed at 4 °C for 15 min for solidification of the agarose layer and subsequently incubated in an inverted manner at 37 °C in an incubator containing 5% CO_2_. After seven days, 2 mL of 4% paraformaldehyde (Avantor, Radnor, USA) in PBS was added to the wells for fixation and again incubated at room temperature for 120 min. Paraformaldehyde solution was then removed, the agarose overlay removed by using a water flow and then cells were stained with 200 μL/well of 0.4% methylene blue solution (Thermo Fisher Scientific) for a few seconds. Finally, wells were gently rinsed with water, dried by inverting the plates on towels, and plaques were counted.

### Infection of human primary airway cells (MucilAir™) with RSV-NICA

3D human nasal epithelial pooled donor MucilAir™ tissues (Batch Number: MP0010; Certificate of Analysis in Additional file 1) were sourced from Epithelix Sàrl (Geneva, Switserland). The tissues were maintained as per the manufacturer’s instruction until the day of infection and treatment. At day 0 of the experimental protocol, the tissues were washed with 0.5 ml MucilAir™ medium and trans-epithelial electrical resistance (TEER) and cilia beating were evaluated. In a separate 24-well plate, 1:10 dilutions of different analysis samples were prepared in MucilAir™ medium by mixing 320 μl/well of MucilAir™ medium with 40 μl of each vial/condition to all relevant wells. Tissues were then infected apically with 100 μl/well RSV-NICA or RSV-A-Long virus and incubated with the virus at 37 °C in 5% CO_2_ for 1.5 h. Non-infected tissues were included as negative controls. The virus was aspirated, the tissues were washed with PBS and apical wash fluid was collected and stored at -80 °C. The tissues were further incubated for 9 days at 37 °C in 5% CO_2_. Cilia beating was evaluated and the tissues were washed with PBS and the apical wash fluids were collected and stored at -80 °C for RT-qPCR analysis. On days 1, 2, 4, and 9 of cell culturing, membrane integrity (TEER) and lactate dehydrogenase (LDH) release was measured to analyze the health status of the tissues. At the end of the experiment, cell viability of tissues was checked with an MTT assay according to manufacturer’s instructions. Epithelix’ protocols were used for evaluation of TEER and LDH.

### Measurement of viral load using RT-qPCR

For RT-qPCR, 5 μl of the apical wash was added to 45 μl of lysis buffer (Cells-to-cDNA II Cell Lysis buffer, Thermo Fisher Scientific). Viral RNA from 150 μl of the RSV-NICA stocks was extracted with the NucleoSpin RNA Virus kit (Macherey–Nagel, Allentown, USA) as per the manufacturer’s instructions. Viral loads were determined by RT-qPCR assay as described below. RT-qPCR was performed using the iTaq™ Universal Probes One-Step Kit (Bio-Rad). Briefly, 2 μl of the extracted viral RNA was mixed with 5 μl of the buffer from the kit, 0,25 μl of the RT enzyme, 60 μM of the forward (CTGTGATAGARTTCCAACAAAAGAACA) and reverse (AGTTACACCTGCATTAACACTAAATTCC) primer for RSV (IDT), 2.5 μM of the probe (FAM-CAGACTACTAGAGATTACC) for RSV (IDT) and 1.55 μl of water to a final volume of 10 μl. Each reaction was performed in duplicate. PCR plates (Roche) were sealed with Lightcycler sealing tape (Roche) and centrifuged for 1 min at 750 rpm. The one-step PCR reaction and subsequent amplification analysis were carried out using the Roche Lightcycler 480 Real Time PCR System using the following conditions: 50 °C for 10 min, 95 °C for 3 min, followed by 40 cycles of qPCR for 15 s at 95 °C and at 60 °C for 1 min. A standard curve was generated using a Gblock (IDT) (Additional file 2) against which the RSV RNA content measured from test samples was quantified (Additional file 3). All Cq values of the RSV samples fall in the linear range of the standard curve.

### Cilia beating analysis

Analysis of cilia beating was performed using the Sisson-Ammons Video Analysis (SAVA) software (Ammons Engineering, Michigan, USA). On each insert two microscopic fields were measured with a 10 × objective on a Zeiss Axiovert microscope (Zeiss, Jena, Germany) with a Basler acA1300-200 μm USB3 video camera. A 5-s video, 100 frames per second, 512 frames was taken and further analyzed with the software. Results are provided as percentage average active area (AAA).

### Determination of barrier integrity

To determine the integrity of the tissue barrier, warm MucilAir™ medium (200 μl) was added on the apical surface of human nasal MucilAir™ tissues and the electrical resistance of the tissues was measured with a Millicell ERS-2 (Merck/Millipore, Darmstadt, Germany) voltohmmeter. After measuring all tissues, medium was gently aspirated from the apical surface of the MucilAir™ inserts.

### Measurement of membrane integrity using LDH assay

The LDH Assay Kit-WST according to Dojindo’s protocol (Tebu Bio, Boechout, Belgium) was used to assess cytotoxicity in MucilAir™ tissues, per manufacturer’s instructions. Briefly, 100 μl of the basolateral medium was used for the LDH assay and replaced by fresh medium. The collected medium was incubated for 30 min with 100 μl LDH substrate mix in a 96-well plate. The reaction was stopped by the subsequent addition of 50 μl stop solution. Fresh MucilAir™ medium incubated with the quantification reagents was used as a background control. Absorbance measurements were done using a multi-mode microplate reader (490 nmBMG Labtech, Ortenberg, Germany).

### Measurement of mitochondrial activity to determine cell viability

Cell viability was analyzed on day 9 of cell culturing using the MTT assay (Merck, USA). Briefly, the conversion of MTT (3-(4,5-dimethylthiazol-2-yl)-2,5-diphenyltetrazolium bromide) tetrazolium salt into its reduced formazan form was assessed. A MTT stock was prepared in Dulbecco’s PBS at a concentration of 5 mg/ml. The MTT substrate was prepared in MucilAir™ medium and added to the basolateral medium at a final concentration of 1 mg/ml and incubated for 2–3 h at 37 °C and 5% CO_2_. The quantity of formazan was measured by recording changes using a multi-mode microplate reader in absorbance mode (570 nm; Clariostar, BMG Labtech). Cell viability was expressed as the percentage of relative absorbance of treated cells relative to the control cells.

## Results

### Assessment of the stability and homogeneity of RSV-NICA viral titer

In 2018, two batches of RSV-NICA stock were produced. From one of these batches, consisting of twelve 96-vial boxes, 48 vials were randomly selected (four per box) and preserved on ice for an indeterminate period before the infectious viral titer of RSV-NICA in each vial was determined (Table 1). A substantial variation in the infectious viral titer was observed among the chosen vials from the distinct boxes, with no detectable infectious virus remaining in vials from boxes VI and VIII (all vials TCID_50_ < 2.35 log_10_) and up to an average TCID_50_ = 5.67 ± 0.07 log_10_ in vials from box X.

Based on these findings, we resolved to re-assess the viral titers of four randomly selected vials from each box that exhibited an average initial TCID_50_ > 4.0 log_10_ in 2018. A total of 24 unique vials (four from each of boxes I, II, III, IV, X and XII) were titered using conventional TCID_50_ determination and plaque assay protocols, as well as RT-qPCR, to thoroughly characterize the five-year stability and homogeneity of the viral titer in the selected RSV-NICA vials. In general, the infectious viral titers of the re-analyzed vials correlated well, regardless of whether they were assessed via TCID_50_ or plaque titration (Additional file 4). In those vials where infectious virus could be detected, the measured infectious titer ranged between 4.17 and 5.83 log_10_ TCID_50_/ml and 2.64 and 5.11 log_10_ PFU/ml, respectively (Table 2). The average infectious titer of RSV-NICA as determined across all 24 vials was 4.17 ± 0.16 log_10_ TCID_50_/ml. Compared to the historical analysis conducted 5 years prior, there was an approximate overall reduction of 0.8 log_10_ TCID_50_/ml observed, from 4.99 ± 0.11 log_10_ to 4.17 ± 0.16 log_10_ TCID_50_/ml. Although this reduction in infectious viral titer was statistically significant, it was not uniformly observed across all vials, but was confined to reductions in only a limited number of vials. The infectious viral titers measured in vials A3, C2, J1, J3, L2 and L3, were substantially reduced or entirely eliminated (Table 2). Vial C4 was excluded from the study due to contamination during the analysis. It is unclear why the infectious viral titers of the abovementioned vials were reduced, but when these vials were removed from the analysis, the current average infectious titer of the remaining vials was essentially identical to the historical titer analyzed five years earlier, i.e. 4.75 ± 0.06 log_10_ TCID_50_/ml.

**Table 2:** (Infectious) viral titers from selected RSV-NICA vials

Sample ID	TCID_50_/ml (log_10_)	Average TCID_50_/ml ± SEM	PFU/ml (log_10_)	Average PFU/ml ± SEM	vRNA copies/ml (log_10_)	Average vRNA copies/ml ± SEM
A1	4.50	4.17 ± 0.56	4.52	3.97 ± 0.45	8.22	8.28 ± 0.05
A2	4.83		4.56		8.26	
A3	2.50		2.64		8.33	
A4	4.83		4.18		8.31	
B1	4.17	4.67 ± 0.17	3.88	4.75 ± 0.29	8.32	8.30 ± 0.02
B2	4.83		5.05		8.27	
B3	4.83		5.11		8.29	
B4	4.83		4.95		8.30	
C1	4.17	3.72 ± 0.62	3.96	2.68 ± 1.14	8.26	8.23 ± 0.08
C2	2.50		1.00		8.18	
C3	4.50		4.76		8.33	
C4			1.00		8.16	
D1	4.83	4.75 ± 0.16	4.49	4.41 ± 0.18	8.31	8.32 ± 0.03
D2	4.50		4.88		8.31	
D3	4.50		4.04		8.37	
D4	5.17		4.24		8.29	
J1	2.50	3.92 ± 0.84	1.00	3.21 ± 1.02	8.27	8.29 ± 0.09
J2	4.83		4.94		8.37	
J3	2.50		1.95		8.17	
J4	5.83		4.95		8.35	
L1	4.50	3.67 ± 0.69	4.48	2.79 ± 1.04	8.31	8.29 ± 0.05
L2	2.50		1.00		8.22	
L3	2.50		1.00		8.28	
L4	5.17		4.70		8.34	

Viral infectious titers that are shown. Lower limit of detection was set at 2.5 log_10_ for TCID50 and at 1 log_10_ for PFU

In contrast to lower the infectious viral titers measured in vials A3, C2, J1, J3, L2 and L3, titration through the quantification of viral RNA did not reveal any significant differences as the titer in all the vials ranged between 8.16 and 8.47 log_10_ vRNA copies/ml (Table 2).

In light of these initial virus titration results, we endeavored to explore whether the homogeneity of an RSV virus stock could be restored via a pooling, re-aliquoting and re-titration procedure. Given that such a procedure is intrinsically linked to introducing an additional freeze–thaw cycle, the impact of such a single or multiple cycle(s) of freeze-thawing on the infectious viral titers was evaluated. Aliquots from the first three vials from boxes J and L, respectively, were pooled and the virus titers from the pools were determined before and after a single freeze–thaw cycle. Despite a lower infectious titer detected in 2/3 of the selected vials (see Table 2), infectious titers in both pooled samples were found to be at least 50-fold above the lower limit of detection as measured via TCID_50_ and plaque assay methods. The average infectious titer of RSV-NICA in pool J before freeze-thawing was 4.50 log_10_ TCID_50_/ml. Importantly, a freeze–thaw cycle did not appear to have a negative influence on the infectious virus titers, since the viral titer of pooled samples J and L were approximately equal to the viral titer determined for the corresponding sample that underwent a freeze-thawing cycle (Table 3).

**Table 3:** Effect of a single freeze–thaw cycle on the infectious viral titer from RSV-NICA

Sample ID	Pool J		Pool L	
Freeze thaw	**Before**	**After**	**Before**	**After**
TCID_50_/ml	**4.50**	**4.50**	**4.17**	**4.17**

Viral infectious titers that are shown. Lower limit of detection was set at 2.5 log_10_ for TCID_50_

To further investigate this, we aimed to assess if the infectious viral titer of the RSV-NICA virus could be maintained after multiple freeze–thaw cycles. Therefore, 30 additional vials of RSV-NICA with varying infectious viral titer (as determined in 2018) were pooled, thoroughly mixed and re-aliquoted in separate vials. On three separate days, all aliquots were thawed and snap frozen again and infectious virus titers in each of the vials were determined before and after each freeze–thaw cycle. The TCID_50_ of the pooled virus before the first freeze–thaw cycle was determined to be 5.19 log_10_ TCID_50_/ml. Five replicate sets of vials were then subjected to multiple separate freeze–thaw cycles and the infectious viral titer of RSV-NICA in the replicate vials was measured. We observed no impact on the infectious viral titer of RSV-NICA as a result of re-aliquoting (Table 4). Similarly, no clear negative impact on the infectious viral titer could be observed after up to three rounds of freeze-thawing as the average TCID_50_ measured was 5.23 ± 0.64 log_10_, 5.09 ± 0.35 log_10_ and 5.12 ± 0.45 log_10_, after a first, second and third round of freeze-thawing, respectively.

**Table 4:** Effect of multiple freeze–thaw cycles on the infectious viral titer from RSV-NICA

Sample ID	Procedure	TCID_50_/ml (log_10_)
Pooled stock	Before aliquoting No freeze–thaw	5,19
Pooled stock	After aliquoting No freeze–thaw	5,19
Aliquot 1	First freeze–thaw	5,02
	Second freeze–thaw	5,02
	Third freeze–thaw	4,84
Aliquot 2	First freeze–thaw	5,19
	Second freeze–thaw	5,02
	Third freeze–thaw	5,02
Aliquot 3	First freeze–thaw	5,19
	Second freeze–thaw	5,02
	Third freeze–thaw	5,19
Aliquot 4	First freeze–thaw	5,02
	Second freeze–thaw	5,02
	Third freeze–thaw	5,37
Aliquot 5	First freeze–thaw	5,72
	Second freeze–thaw	5,37
	Third freeze–thaw	5,19

Infection and replication potential of RSV-NICA in human primary airway cells

To ascertain the ability of RSV-NICA to infect its human target cells [3, 21], virus from each of three vials from boxes A, B, C, D, J and L were used to infect pooled MucilAir™ nasal epithelial tissues. Furthermore, aliquots of three vials from boxes J and L, respectively, were pooled and the virus titers from the pools were determined using RT-qPCR before and after a freeze–thaw cycle.

**Fig. 1:**
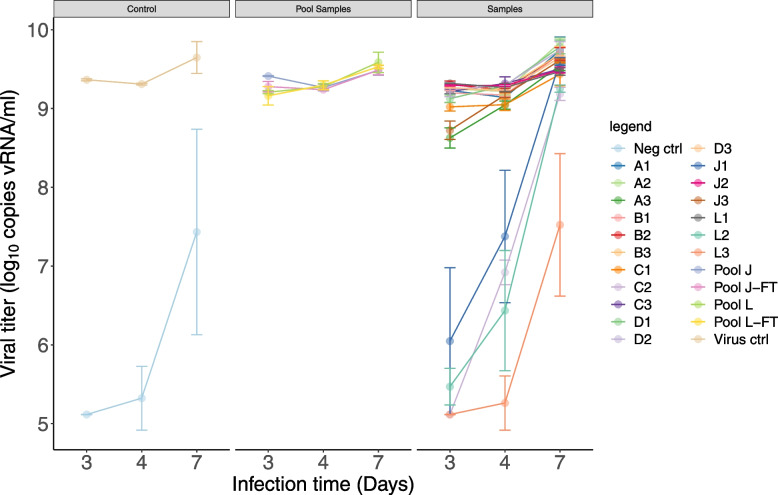
Assessment of infectivity of RSV-NICA vials in MucilAir™. The viral titer determined by RT-qPCR in the apical compartment of air–liquid interface cultures of human nasal epithelial cells on day 3, 4 and 7 post-infection is expressed as vRNA copies/ml (log_10_). For each vial, the result of one independent infection experiment is displayed and presented as the mean of 3 technical replicates (colored dots) with standard error. Aliquots of the 3 vials from boxes J and L, respectively, were pooled and the virus titers from the pools were determined before and after a freeze–thaw cycle. RSV Long-A was included as a positive virus control. Negative control did not contain RSV, except in 2 out of 3 tissue replicates at day 7 after infection due to contamination of the tissues (by aerosol or pipetting). The day 7 results were not used for evaluation

Infection of nasal epithelial cells was observed with RSV-NICA present in each of the selected vials (Fig. 1). Moreover, RSV-NICA replicated in each of the infected cells. In cells infected with virus from vials A3, C2, J1, J3, L2 and L3, a lower copy number of vRNA was observed as compared to the copy number in the other vials, fully corresponding with the lower infectious viral titers observed in these vials. Again, the freeze–thaw cycle did not appear to impact the infectivity and capacity of RSV-NICA to replicate in human primary nasal epithelial cells as similar infection and viral replication was observed in the pooled vials from boxes J and L before and after the freeze–thaw cycle.

### Functional effect of RSV-NICA infection on human primary airway cells

Prior to infection with RSV-NICA, the barrier integrity of Mucilair™ tissues was measured (Additional file 5). All tissues were found to be in a healthy state with TEER values > 200 Ohm.cm^2^. TEER values were measured again on day 9 post-infection and found to be equal to those measured before infection. These results indicate that infection with RSV-NICA did not impact barrier integrity of the cell tissues. Concurrent with a maintained barrier integrity, no decrease in mean cell viability (% viability > 85%) or LDH detected in the supernatant up to day 9 post-infection was observed when Mucilair™ tissues were infected with RSV-NICA (Additional file 6).

**Fig. 2:**
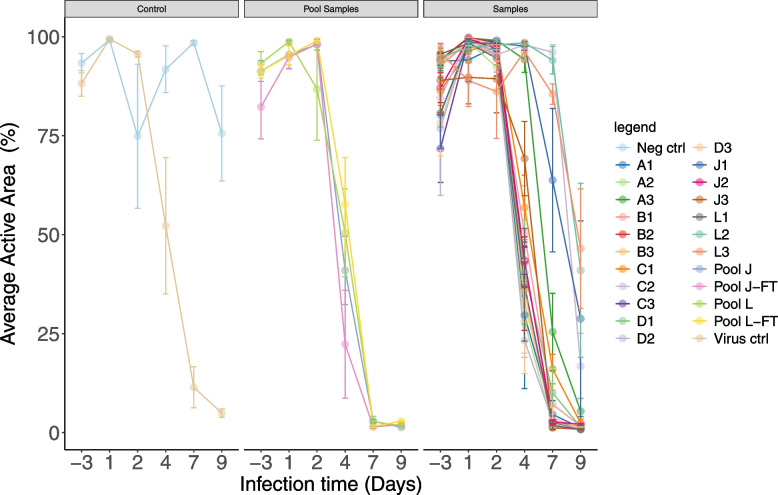
Impact of RSV-NICA infection on cilia beating. Assessment of cilia beating (as percentage average active area, AAA) was performed 3 days before infection (Days-3) and on days 1, 2, 4, 7 and 9 after infection. For each vial, the result of one independent infection experiment is displayed and presented as the mean of 3 technical replicates (colored dots) with standard error. Aliquots of the 3 vials from boxes J and L, respectively, were pooled and the cilia measurement from the pools were determined before and after a freeze–thaw cycle. RSV Long-A was included as a positive virus control. Negative control did not include RSV

To determine if infection and replication of RSV-NICA has a negative effect on the function of its ciliated epithelial target cells, the cilia beating of these cells was measured. A clear reduction of cilia beating was found in function of time after infection, with little to no effect on cilia beating on day 1 and 2 after infection, but with an increasingly noticeable reduction of cilia beating from day 4 post-infection (Fig. 2). A delayed reduction of cilia beating was observed when cells were infected with virus from vials A3, C2, J1, J3, L2, and L3, again correlating with the lower infectious titer found in these vials as well as with the delayed viral replication in cells infected with virus from these wells (Fig. 1). Cilia beating was also reduced from day 4 post-infection in the pooled vials from boxes J and L, concomitant with the reduction observed with RSV-NICA from the individual vials. No additional impact of a freeze–thaw cycle on cilia beating could be observed as the reduction in cilia beating was equal in time and extent when virus from pooled RSV-NICA before and after the freeze–thaw cycle was used to infect human airway epithelial cells.

## Discussion

CHIM studies hold the potential to provide developers with a comprehensive understanding of the mechanism-of-action and prospective efficacy of novel anti-infective preventive or treatment strategies during the initial stages of development. This facilitates informed decision making regarding prioritizing of biopharmaceutical modalities. The successful conduct of CHIM studies necessitates the availability of GMP-qualified challenge agents that preferably display long-term stability and homogeneity in their (infectious) viral titer. In 2018, SGS Belgium NV developed RSV-NICA, an RSV type A challenge agent intended for use in RSV CHIM studies. Given the known susceptibility of RSV infectivity to various environmental factors such as temperature and pH [2, 7, 5, 8, 11], we sought to re-analyse the (infectious) viral titers and infectivity of RSV-NICA. This paper reports the findings of a study investigating the five-year stability and homogeneity of RSV-NICA, and discusses potential avenues for its long-term utilization as an RSV challenge agent.

In a first series of experiments, the (infectious) viral titer of RSV-NICA challenge agent was determined through various titration methodologies. For this purpose, a set of 24 distinct vials (4 randomly chosen from each of the 6 boxes containing 96 vials), which had previously been assessed in 2018 to possess infectious viral titers of > 4.0 log_10_ TCID50/ml, were subjected to viral re-titration. The infectious viral titer of the selected vials was determined via two independent methods, namely TCID_50_ and plaque assay analyses, revealing the presence of infectious virus in the majority of the vials with titers ranging between 4.17 and 5.83 log_10_ TCID_50_/ml and 2.64 and 5.11 log_10_ PFU/mL. A strong correlation (*r* = 0.94) was noted between the viral infectious titers as measured by the two different methodologies, indicating the robustness of the analysis. Compared to the infectious titer of RSV-NICA as determined five years prior, the average titer across the 24 vials measured in this study exhibited a significant reduction, approximately 0.8 log_10_ TCID_50_/ml. However, this reduction in titer was not attributable to a general decrease across all vials, but was due to a significant decrease in a limited number of vials. Specifically, vials A3, C2, J1, J3, L2 and L3 displayed a significantly reduced infectious viral titer or a titer below the lower limit of detection. Upon exclusion of these vials from the calculation, a comparable infectious viral titer was obtained between the recent and the historical analysis (4.75 ± 0.06 vs 4.99 ± 0.11 log_10_ TCID_50_/ml; *p*-value = 0.14).

The RT-qPCR analysis of RSV-NICA RNA demonstrated a consistent presence in all samples (8.16–8.47 log_10_ vRNA copies/ml), inclusive of vials A3, C2, J1, J3, L2 and L3. This data suggests that equal amounts of virus were likely aliquoted following the fresh GMP-production of the RSV-NICA stock, but the infectivity of the virus in some of the individual vials may have been adversely impacted during the five-year storage period and/or by variable incubation time on ice upon thawing.

Consistency in infectious viral titer across all vials of a challenge agent batch is a critical factor when conducting CHIM studies, as occasional variations in these titers pose potential challenges in terms of patient safety as well as scientific and operational aspects. Given the occasional inconsistencies observed in the infectious viral titer of the virus present in individual vials, we contemplated whether pooling—along with the inherent introduction of additional freeze–thaw cycles – and re-titration could serve as an effective strategy to ensure better control over the consistency in infectious viral titer across vials of a challenge agent batch over time. An initial experiment involving the introducing of a single freeze–thaw cycle did not result in to a loss of infectious virus, providing preliminary evidence that pooling, re-titration and subsequent freezing could present a means to maintain a sufficiently high infectious viral titer while restoring the homogeneity of the titer, thereby extending the shelf-life of an RSV challenge agent. Furthermore, we examined the stability of the infectious viral titer of the RSV-NICA challenge agent following exposure to multiple freeze–thaw cycles. Again, no noticeable impact on the infectious titer of the virus was observed, further substantiating the potential of pooling as a strategy to prolong the shelf-life of RSV challenge virus to at least the period of one CHIM trial and facilitates its flexible application in CHIM studies. Future follow-up is required to determine long time stability. The stability of the infectious viral titer of the virus in a vial after multiple freeze–thaw cycles allows for the use of single vials in smaller size CHIM trials where participants need to be inoculated over several days.

In the second series of experiments, we aimed to investigate the ability of RSV-NICA virus in a set of selected vials to infect its primary human target cells and its capacity to replicate in these cells. MucilAir™ cells were infected with RSV-NICA and infection and replication were assessed on different days post-infection by quantifying the amount of vRNA in the cells. Additionally, the effect of the virus on cell viability and functionality was evaluated. The nasal epithelial cells were infected with RSV-NICA present in all tested vials and approximately equivalent levels of vRNA were detected, except when cells were infected with virus from vials A3, C2, J1, J3, L2 and L3. For these vials, replication in ciliated epithelial cells was delayed compared to the other vials, consistent with a lower titer of infectious virus found in these vials.

Infection of MucilAir™ cells with RSV-NICA did not appear to significantly reduce cell viability/cytotoxicity or compromise the integrity of the membrane barrier function of the nasal epithelial tissues. This could potentially be attributed to the fact that RSV displays tropism for only the ciliated cells, a subset of cells in the nasal epithelial tissues [3, 21]. However, infection with RSV-NICA did result in a reduction of cilia beating correlating with time post-infection. Analyzed on days 3, 4 and 7 post-infection, an equaivalent reduction of cilia beating was induced in the cells when infected with virus from all vials, even with virus present in vials A3, C2, J1, J3, L2 and L3, although delayed and consistent with the lower infectious viral titer of these vials.

Our findings are intriguing and underscore the high potential of RSV-NICA as a CHIM challenge agent. With infectious viral titers of RSV-NICA ranging between 4.17 and 5.83 log_10_ TCID_50_/ml or 2.64 and 5.11 log_10_ PFU/mL across different vials of the virus batch, and with a stable infectious viral titer in an estimated 75% of the vials (18/24 vials in our analysis), it can be anticipated that the majority of the vials in the virus batches could be utilized in subsequent CHIM studies over a period of at least 5 years. In a recent human challenge study evaluating the efficacy of EDP-938, a small-molcule RSV N-protein inhibitor, study participants were inoculated with 0.8 ml of viral inoculate, corresponding to approximately 4.0 log_10_ PFU/ml [1]. During the first part of the study, 115 participants were enrolled, while during the second part, 63 subjects were quarantined, inoculated, and randomly assigned to a trial group. Of these, 86 and 38 participants, respectively, were included in the intention-to-treat-infected analysis, demonstrating the high inoculation efficiency across the study groups. Since we also demonstrated that subsequent cycles of pooling and re-aliquoting including freeze-thawing of RSV-NICA, do not appear to influence its infectious titer in these amount of vials needed for one CHIM trial, such a procedure can be employed to maintain a stable and homogeneous infectious viral titer across the stock of challenge agent, thereby further extending its shelf-life and minimizing patient safety, scientific, and operational challenges associated with study participant inoculation during RSV CHIM studies.

## Conclusion

Collectively, our data demonstrate that the infectious viral titer of our RSV-NICA challenge agent can be maintained over a multi-year time period, although sporadic loss of infectivity in individual vials may occur. The data presented herein further suggest that pooling and re-titration is a viable strategy to further extend the shelf-life of the challenge agent to at least the period of one CHIM trial. Consequently, operational aspects of viral inoculation of the trial participants are optimized, enhancing the safety of the participants. This further demonstrates that RSV-NICA is a stable, suitable CHIM agent that can be used in efficacy trials for RSV vaccines and antiviral entities. A human titration study will be conducted to finalize this RSV Controlled Agent model.

### Supplementary Information

Supplementary Material 1.

Supplementary Material 2.

Supplementary Material 3.

Supplementary Material 4.

Supplementary Material 5.

Supplementary Material 6.

## Data Availability

No datasets were generated or analysed during the current study.
