# Supplementary figures and images for: Proof of stability of an RSV Controlled Human Infection Model challenge agent
Source: Virol J. 2024 May 15;21:112. doi: 10.1186/s12985-024-02386-y (PMC11097566; doi:10.1186/s12985-024-02386-y)

# Additional file 1

**Certificate of Analysis**


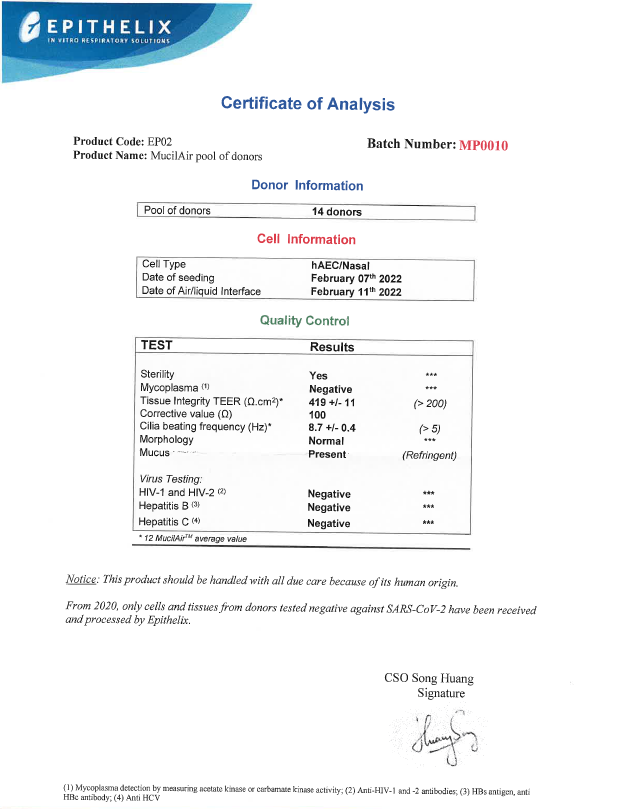

Supplement: Supplementary file 1 — Supplementary Material 1. [file 12985_2024_2386_MOESM1_ESM.docx]
