# Supplementary material for: Proof of stability of an RSV Controlled Human Infection Model challenge agent
Source: Virol J. 2024 May 15;21:112. doi: 10.1186/s12985-024-02386-y (PMC11097566; doi:10.1186/s12985-024-02386-y)
Supplement: Supplementary file 2 — Supplementary Material 2. [file 12985_2024_2386_MOESM2_ESM.docx]

# Additional file 2


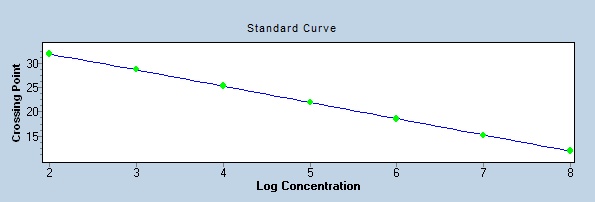


**Additional file 2: Standard curve of Gblock.** A series of dilutions of the Gblock was prepared (100 copies/ml (i.e. Log Concentration 2) up to 1E+08 copies/ml (i.e. Log Concentration 8). The Crossing Point (Cp) values were measured for each dilution.
