# Supplementary material for: Proof of stability of an RSV Controlled Human Infection Model challenge agent
Source: Virol J. 2024 May 15;21:112. doi: 10.1186/s12985-024-02386-y (PMC11097566; doi:10.1186/s12985-024-02386-y)
Supplement: Supplementary file 3 — Supplementary Material 3. [file 12985_2024_2386_MOESM3_ESM.docx]

# Additional file 3


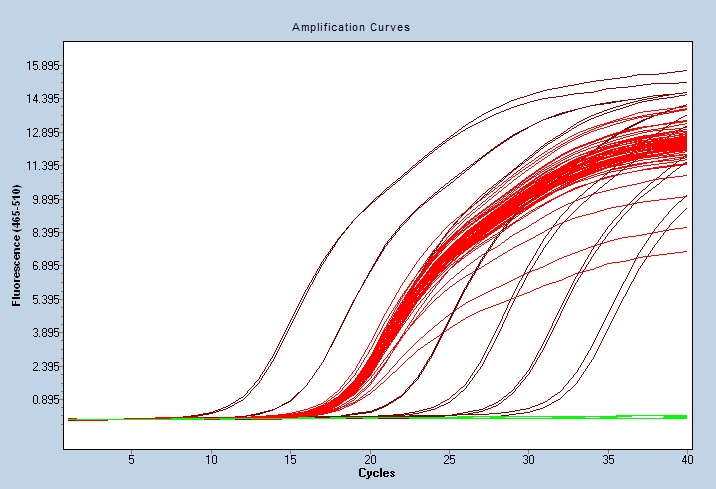


**Additional file 3: Amplification curves of Gblock (dark red) and RSV samples (bright red).** The RSV RNA content in the samples was quantified by comparing their Cq values to the standard curve. All Cq values of RSV samples fall in the linear range of the standard curve. The Cq value of the RSV samples corresponds to a specific concentration of RSV RNA based on the standard curve (Additional file 2).
