# Supplementary material for: Proof of stability of an RSV Controlled Human Infection Model challenge agent
Source: Virol J. 2024 May 15;21:112. doi: 10.1186/s12985-024-02386-y (PMC11097566; doi:10.1186/s12985-024-02386-y)
Supplement: Supplementary file 4 — Supplementary Material 4. [file 12985_2024_2386_MOESM4_ESM.docx]

# Additional file 4


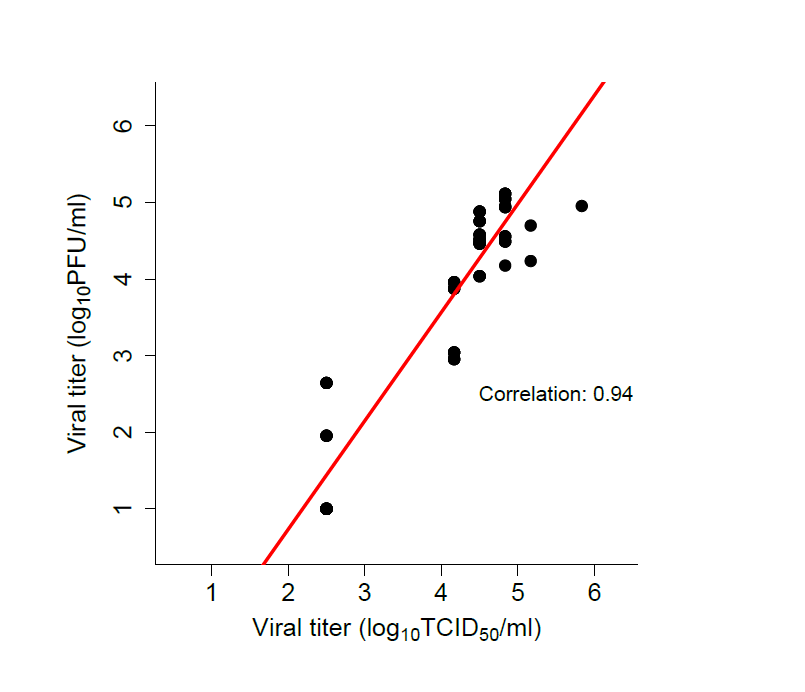


**Additional file 4: Correlation between infectious viral titers from RSV-NICA as analysed by two different methods.**
