# Supplementary material for: Proof of stability of an RSV Controlled Human Infection Model challenge agent
Source: Virol J. 2024 May 15;21:112. doi: 10.1186/s12985-024-02386-y (PMC11097566; doi:10.1186/s12985-024-02386-y)
Supplement: Supplementary file 5 — Supplementary Material 5. [file 12985_2024_2386_MOESM5_ESM.docx]

# Additional file 5

A B
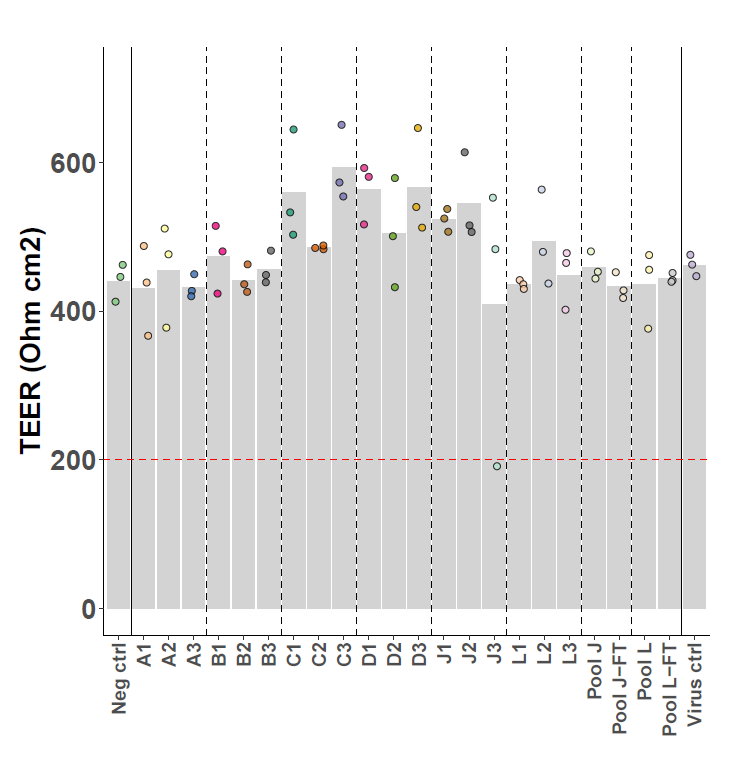

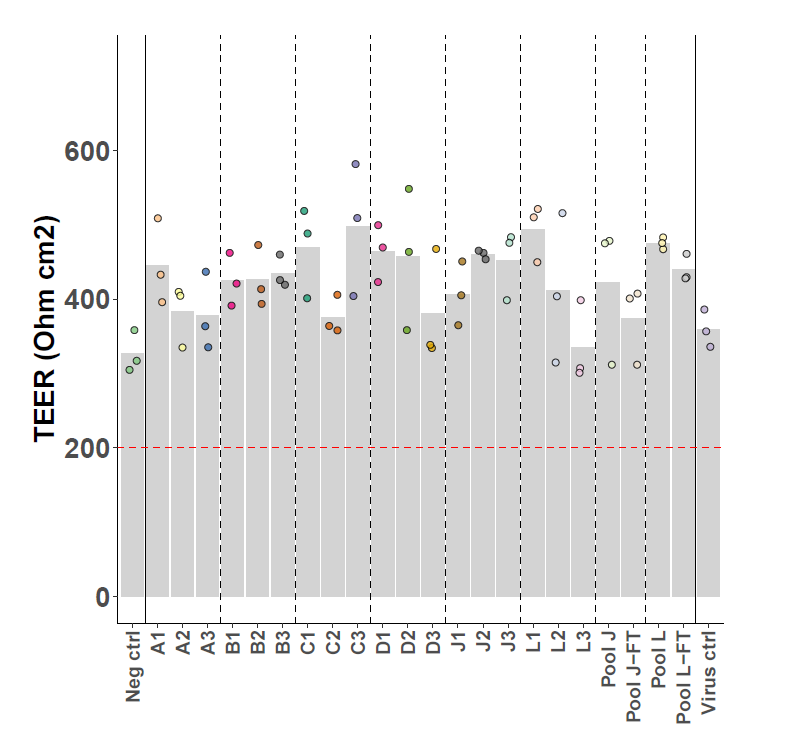


**Additional file 5: Measurement of barrier integrity (TEER, Ohm.cm²) in MucilAir™ 3 days before infection (A) and 9 days after infection (B) with different RSV-NICA aliquots.** Aliquots of the 3 vials from boxes J and L, respectively, were pooled and barrier integrity from the pools were determined before and after a freeze-thaw cycle. RSV Long-A was included as a positive virus control. Negative control did not include virus.
