# Supplementary material for: Proof of stability of an RSV Controlled Human Infection Model challenge agent
Source: Virol J. 2024 May 15;21:112. doi: 10.1186/s12985-024-02386-y (PMC11097566; doi:10.1186/s12985-024-02386-y)
Supplement: Supplementary file 6 — Supplementary Material 6. [file 12985_2024_2386_MOESM6_ESM.docx]

# Additional file 6

| **Sample ID** | **% Cell viability (MTT)** | **Sample ID** | **% Cell viability (MTT)** |
| --- | --- | --- | --- |
| **Negative control** | 100 | **D3** | 86 ± 0.09 |
| **A1** | 99 ± 1.82 | **J1** | 99 ± 2.81 |
| **A2** | 98 ± 6.15 | **J2** | 92 ± 11.04 |
| **A3** | 92 ± 9.46 | **J3** | 102 ± 7.54 |
| **B1** | 90 ± 7.23 | **L1** | 102 ± 12.55 |
| **B2** | 89 ± 5.37 | **L2** | 100 ± 3.25 |
| **B3** | 87 ± 7.42 | **L3** | 97 ± 11.40 |
| **C1** | 101 ± 4.36 | **Pool_Freezethaw J** | 102 ± 9.83 |
| **C2** | 102 ± 5.56 | **Pool-Freezethaw L** | 94 ± 10.90 |
| **C3** | 85 ± 2.95 | **Pool J** | 90 ± 1.05 |
| **D1** | 87 ± 6.63 | **Pool L** | 92 ± 11.07 |
| **D2** | 81 ± 2.73 | **Virus Control** | 96 ± 11.51 |

**Additional file 6: % cell viability (MTT) in MucilAir™ after infection with RSV-NICA.**
